# Fixation of metatarsal fracture with bone plate in a dromedary heifer

**Published:** 2013-03-02

**Authors:** S.A. Siddiqui, M.I. Siddiqui, M.N. Telfah, S. Hashmi

**Affiliations:** 1 *Veterinary Hospital, Al-Qattara, Alain, United Arab Emirates*; 2 *Central Veterinary Hospital, Al-Wathba, Abu Dhabi, United Arab Emirates*

**Keywords:** Bone plating, Camel, Fracture, Interfragmental compression, Metatarsus

## Abstract

An oblique fracture of the distal third of the right metatarsus in a three-year-old dromedary heifer weighing about 300 kilograms was immobilized with a 4.5 mm broad-webbed 12-hole dynamic compression bone plate and two interfragmental compression screws. The animal showed slight lameness after 16 weeks of surgery that disappeared after removal of the plate. The result was quite encouraging and the fracture healed in 16 weeks without major complications. It is concluded that the fracture of this bone can be successfully handled with bone plating at least in young, light weight animals.

## Introduction

The only remaining metatarsal rays in the camel are metatarsal-3 and metatarsal-4 which are fused proximally. The distal aspect diverges to form independent articulations with the third and fourth digits. Causes of fractures of long bones in animals are manifold; the main being the road accidents, falling during exercise, violent impact against a fixed object or due to bone pathology when the fracture occurs even under slight stress (Ramadan and Al-Mubarak, 2007; Tuttle *et al.*, 2007).

The fractures can be classified on different basis and each fracture has its own dynamics (Müller, 1991; Ramadan, 1992). Methods of internal fixation of fractures in small animals are a commonplace, but these methods still have certain limitations in the large animals. Different methods of external and internal fixation of long bone fractures have been described in llamas (Jean *et al.*, 1989), llamas and small ruminants (Kaneps *et al.*, 1989), camelids (Tee *et al.*, 2005; Ramadan and Al-Mubarak, 2007) and llamas and alpacas (Newman and Anderson, 2007; Newman and Anderson, 2009).

A comprehensive discussion on complications after orthopedic surgery in alpacas has been reported by Semevolos *et al*. (2008). Wilson and Vanderby (1995) have evaluated application of fiberglass cast to immobilize long bone fractures in large animals. The metacarpal and metatarsal fractures are generally open due to poor muscle mass around these bones, and hence, their immobilization with casts is accompanied with certain disadvantages; the main being that one is unable to see what is happening under the cast. Keeping a window in the cast against the wound allows periodic dressing and evaluation of the fracture site, but it can serve as an open gate for infectious agents and undoubtedly represents the weakest point of the cast (Wilson and Vanderby, 1995). Another limitation to immobilize the metatarsal fracture with the cast in the camel is the sitting posture of this animal. Incorporation of the hock joint in the cast is mandatory in metatarsal fracture and this does not allow the animal to sit in the normal sternal recumbency. Thus repair of fractures of long bones in camels appears difficult using the routine application of plaster casts. In this paper, we report a case of fracture of metatarsal bone in a dromedary heifer that was successfully repaired using bone plating.

## Case Details

A three-year-old ‘Arabian breed’ dromedary heifer weighing about 300 kilograms, suffering from open fracture of the right metatarsal bone was presented to the Central Veterinary Hospital, Al-Wathba, Abu Dhabi, United Arab Emirates. The case had already been handled by a field veterinarian two days earlier, who immobilized the fracture with a Plaster of Paris cast (Gypsona Bandage).

**Fig. 1:**
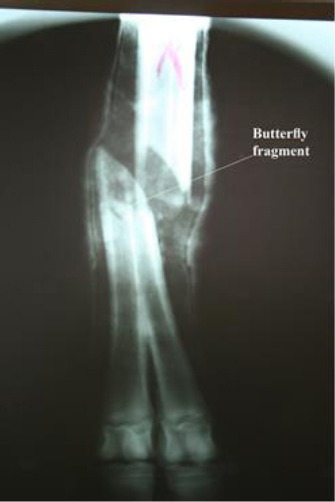
Preoperative dorso-plantar radiograph of the dromedary heifer’s metatarsus.

A dorso-plantar radiograph with the cast still on, revealed an oblique fracture of the distal third shaft of the bone, proximal to the bifurcation with a small butterfly fragment (Fig. 1). Based upon the type and site of the fracture, it was decided to immobilize it with a dynamic compression bone plate.

### Control and Anesthesia

The animal was anesthetized with Isoflurane gas anesthesia mixed with Nitrous Oxide and Oxygen as per procedure described by Murray (2010) and was restrained in the left lateral recumbency keeping the fractured leg on the upper side.

### Operative Procedure

The operative site was prepared for aseptic surgery and the leg was draped. A skin incision was given on the lateral aspect of the metatarsal region starting distally from the hock joint up to above the fetlock joint to expose the fracture site. The fracture was reduced and held with the bone holding clamp. Interfragmental compression was achieved through two 4.5 mm cortical screws in cranio-caudal direction. These screws were used in the lag effect as described by (Smith *et al.*, 1996; Siddiqui and Telfah, 2010).

The near cortex was drilled with a 4.5 mm drill bit using a 4.5 mm tap sleeve. A drill sleeve with an external diameter of 4.5 mm and an internal diameter of 3.2 mm was inserted into the hole until it met the far cortex. The far cortex was drilled using a 3.2 mm drill bit. The drill sleeve was then taken out and the length of the required screw was measured with a depth gauge. The far cortex was tapped out using a 4.5 mm cortex tap. A countersink was cut in the near cortex for the head of the screw. The screw was then driven in and tightened. As the screw took hold only in the far cortex, it resulted in interfragmental compression when tightened.

**Fig. 2:**
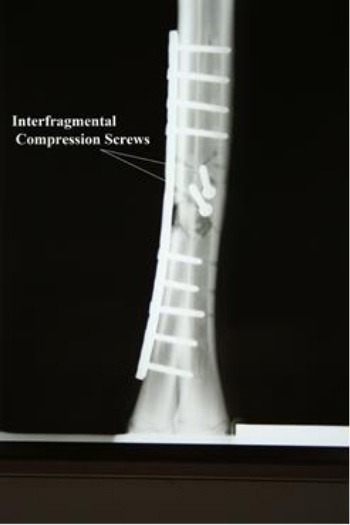
Immediate postoperative dorso-plantar radiograph (Note the placement of the plate and interfragmental compression screws).

Two very small free floating bone pieces were removed, as they had no soft tissue attachment. The butterfly fragment was placed in its proper position. A 4.5 mm broad-webbed 12-hole dynamic compression bone plate was contoured according to the curvature of the bone and applied on its lateral cortex as a neutralizing plate using neutral drill guide (Fig. 2). Five screws in the distal and four screws in the proximal fracture fragment were used, as the middle three screws - if used - would go through the fracture line which could lead to nonunion of the fracture (Semevolos *et al.*, 2008). The subcutaneous tissue was opposed with continuous suture line using USP-1 polyglycolic acid suture material (Safil, B│BRAUN, Aesculap AG & Co. Germany) and the skin edges were sutured with USP-2 braided Supramid suture material (Supramid, B│BRAUN, Aesculap AG & Co. Germany).

### Postoperative Care

A light, polyester-polyurethane bandage (Yoorim Corporation, Korea) was applied from foot to just below the hock joint as a supplemental support. The animal was confined alone in a capacious enclosure away from the other animals and was administered an intramuscular injection of Ampidexlone (Coophavet, France) at the recommended dose rate of 1ml/10kg bodyweight daily for 10 days.

The polyester-polyurethane bandage was removed after two weeks to check the condition of the wound. The suture line was perfectly dry with no signs of infection. The limb was again wrapped in a light polyester-polyurethane bandage for another period of two weeks after which the bandage and the skin sutures were removed.

The owner was advised to keep the animal in a separate enclosure for a further period of eight weeks after which she was allowed free movement. The follow up radiographs were taken 2, 4, 8 and 16 weeks after surgery to evaluate the position of the plate and screws and the healing process.

Although there was radiographic evidence of a bridging callus in the last x-ray picture (16 weeks post-operative), yet to be on the safe side, the plate was removed 24 weeks after surgery. The interfragmental screws could not be removed, as they were fully covered with the callus. At the time of removal of the plate, the screw at the extreme end of the plate in the distal fracture fragment was found loose.

## Discussion

**Fig. 3:**
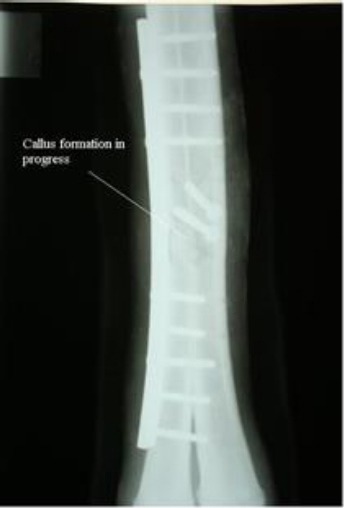
Dorso-plantar radiograph (eight weeks post-operation). Callus formation in progress.

**Fig. 4:**
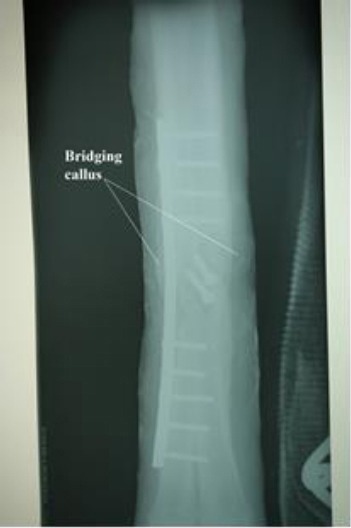
Dorso-plantar radiograph (16 weeks post-operation). Note the bridging callus.

The animal recovered from injury without major complications except slight lameness after 16 weeks of surgery, but was able to bear full weight on the affected leg when the forelimb of the same side was picked up. The radiographic pictures two and four weeks after surgery revealed that the plate and screws were in place while radiographic examination eight weeks after surgery was indicative of good progress of callus formation (Fig. 3). The radiograph 16 weeks after surgery was indicative of a bridging callus (Fig. 4).

The lameness gradually disappeared after removal of the plate as also recorded previously (Jean *et al.*, 1989; Tee *et al.*, 2005; Newman and Anderson, 2007). The slight lameness was probably due to pressure of the callus on the surrounding tissue and loosening of the last screw in the distal fracture fragment. Similar possibilities have been previously mentioned for llamas and alpacas (Semelovos *et al.*, 2008; Newman and Anderson, 2009). Interfragmental compression resulted in good reduction of the fracture fragments and therefore, healing occurred without complications (Smith *et al.*, 1996; Siddiqui and Telfah, 2010).

In our opinion, factors such as young age of the animal, aseptic surgical procedure, provision of additional support to the fractured limb with Polyester-Polyurethane bandage for the first four postoperative weeks and keeping the animal in a separate enclosure with restricted activity played an important role towards smooth healing of the fracture as also described by Newman and Anderson (2009) and Siddiqui and Telfah (2010).

The animal was having normal gait and locomotion 32 weeks after initial surgery. The results indicated that such fractures can be successfully handled with bone plates if all the precautions to carry out an aseptic surgery are strictly adhered to. Similar views have been previously expressed for repair of long bone fractures in South American camelids (Tee *et al.*, 2005).
